# 外泌体在生物标志物研究领域的文献计量学分析

**DOI:** 10.3724/SP.J.1123.2025.01025

**Published:** 2025-05-08

**Authors:** Yuxia HUANG, Haiyan WANG, Yihan ZHANG, Yifei LIN, Xiaoqiang QIAO, Lianghai HU

**Affiliations:** 1.吉林大学生命科学学院, 吉林 长春 130012; 1. School of Life Sciences, Jilin University, Changchun 130012, China; 2.河北大学药学院, 河北 保定 071002; 2. College of Pharmaceutical Sciences, Hebei University, Hebei 071002, China

**Keywords:** 外泌体, 生物标志物, 液体活检, 疾病诊断, 文献计量学分析, exosomes, biomarkers, liquid biopsy, disease diagnosis, bibliometric analysis

## Abstract

外泌体是细胞分泌的细胞外囊泡的一种亚型,携带丰富的遗传物质及蛋白质等,外泌体膜表面有众多母细胞特有的标志物,已成为疾病诊断、疾病进展以及疾病治疗的重要依据。本文在Web of Science core collection(SCI-EXPENED)数据库中,检索2010-2024年间研究主题为“外泌体”与“生物标志物”或“诊断”或“液体活检”的研究型和综述类论文。文献计量分析结果显示,外泌体作为生物标志物标记疾病的研究关注度逐渐升高,中国是该研究的主要贡献国家;文献主要围绕癌症、炎症、糖尿病、神经退行性疾病和心血管疾病的标记诊断展开研究;通常使用色谱、质谱、拉曼光谱等技术分析外泌体核酸、蛋白质、代谢物等;常用的检验样本为血浆、血清、尿液、唾液、脑脊液等体液;外泌体作为肿瘤标志物的研究主要聚焦于肺癌、乳腺癌、前列腺癌等8种具有高发病率的癌症。本文聚焦于外泌体作为疾病生物标志物的研究领域,利用文献计量工具系统分析了2010-2024年间外泌体及其内容物作为生物标志物在疾病诊断领域中的应用,并对外泌体介导的疾病诊断和疾病治疗的发展前景和未来努力方向进行了分析和展望,期望为外泌体在疾病标志及应用方面的研究提供参考。

## Abstract (en)

Exosomes are extracellular vesicles secreted by cells and are rich in genetic material and proteins. The surfaces of exosome membranes contain many blast-specific markers that provide an important basis for disease diagnosis, progression, and treatment. Herein, we searched the Web of Science core collection (SCI-EXPENED) database for research and review articles on “exosomes” and “biomarkers” or “diagnostics” or “liquid biopsy” as research topics between 2010 and 2024. Bibliometric analysis revealed that exosomes have received increasing levels of attention as disease biomarkers, with China contributing the most to these studies. Herein, we focus on marker diagnoses for cancer, inflammation, and diabetes, as well as neurodegenerative and cardiovascular diseases. Chromatography, mass spectrometry, Raman spectroscopy, and other techniques are typically used to analyze exosomal nucleic acids, proteins, and metabolites, with commonly used test samples including plasma, serum, urine, saliva, cerebrospinal fluid, and other bodily fluids. Research into exosomes as tumor markers has mainly focused on eight highly prevalent cancers, including lung, breast, and prostate cancers. This paper focuses on exosomes as disease biomarkers and uses a bibliometric tool system to analyze the use of exosomes and their contents as biomarkers in the disease diagnosis field between 2010 and 2024, analyzes development prospects, and discusses future exosome-mediated efforts for diagnosing and treating diseases, and is expected to provide a reference for studying and applying exosomes as disease markers.

外泌体(exosome)是直径30~150 nm的纳米级细胞外囊泡(extracellular vesicles, EVs),广泛分布于各类体液中,如血浆、尿液、唾液、脑脊液(cerebrospinal fluid, CSF)以及乳汁等^[1]^。作为细胞间信息传递的重要载体,外泌体携带有丰富的生物分子,如蛋白质、微小RNA(miRNA)、信使RNA(mRNA)、DNA、脂质等^[2]^。这些生物分子能够反映源细胞的病理生理状态,因此,外泌体被认为是最有潜力的生物标志物之一,广泛应用于癌症、心血管疾病、神经退行性疾病、免疫性疾病等的诊断^[3,4]^。例如,利用外泌体核酸(microRNA、mRNA和长链非编码RNA(lncRNA)等)的分布差异来标记特异癌症^[5]^;外泌体蛋白质组学差异分析辅助多种疾病的生理病理状态分析^[6]^;脑脊液tau蛋白磷酸化程度在临床神经退行性病变筛查中也有很大的发展潜力,目前处于实验室到临床的阶段^[7]^。相比于传统的组织活检,外泌体作为液体活检的标志物,由于其易于获得、无创采集且可提供实时的疾病信息,已成为疾病早期诊断和预后评估的重要工具^[8⇓-10]^。在液体活检中使用循环肿瘤DNA(ctDNA)追踪癌细胞的扩散已经成为常见的检测技术^[11]^。2010年,Kosaka等^[12]^研究揭示了外泌体microRNA的分泌机制并发现其在细胞间转移影响癌细胞生长,提出以外泌体microRNA作为生理和病理信号分子的可能;2011年,国际细胞外囊泡学会(ISEV)成立;2013年诺贝尔生理学奖授予James E. Rothman、Randy W. Schekman和Thomas C. Südhof 3位科学家,表彰他们揭示了细胞囊泡运输的调节机制。可见,2010年已有学者关注到外泌体的疾病标志作用,2013年外泌体运输调节机制的阐明推动了研究从理论框架搭建向实践应用转变。因此,本文将2010年作为文献计量分析时间的起始点,以“外泌体”“细胞外囊泡”“生物标志物”等为关键词,利用文献计量学的方法对2010-2024年间外泌体作为生物标志物的研究进展做整理,并结合文献计量学工具对搜索结果进行分析,旨在全面了解外泌体及其内容物作为生物标志物在疾病诊断中的应用及发展情况,为外泌体在疾病诊断研究领域的学者提供数据参考。

## 1 材料与方法

### 1.1 文献检索

本文以Web of Science core collection (SCI-EXPENED) (WOSCC(SCI-E))作为检索数据库,检索式为“(TS=(exosome* OR EV OR extracellular vesicle*)) AND TS=(biomarker* OR diagnostic* OR liquid biopsy)”,时间跨度为2010-01-01至2024-12-31,于2025年1月2日完成文献检索收集的工作,共检索到16951篇文献,文献类型选择“Article”和“Review Article”,排除撤稿文献、重复文献之后得到检索结果为16674篇文献。

### 1.2 研究方法

采用Windows Office Excel 2016、Cite Space V.6.3.R1(64bit)、VOSviewer 1.6.20、Origin 2024等软件对数据进行统计分析和可视化。使用Cite Space对发文年份、关键词突现、关键词时间线图谱做分析。使用VOSviewer对发文量与引用频次的国家和机构分布、发文量与引用频次的期刊分布、发文量与引用频次的作者分布、共被引参考文献和关键词词频做分析。结合数据分析结果,进一步了解外泌体作为生物标志物的研究情况和未来发展趋势。

## 2 结果与讨论

### 2.1 发文年份的分布

特定时间段内的发文量数据可以反映某领域的研究情况和发展趋势。1987年,Johnstone等^[13]^将细胞外的小囊泡命名为“exosome”(外泌体); 2002年科学家们已经注意到血浆蛋白质组学分析具有优越的诊断前景^[14]^;2004年,Pisitkun等^[15]^采集正常人尿液做蛋白质组学分析,确定了几种已有研究证实与肾脏特定疾病或血压调节有关的蛋白质,并提出将尿外泌体蛋白质组学分析作为早期肾脏疾病检测工具的观点,由此开创了外泌体作为疾病生物标志物的研究。2004-2009年间关注外泌体与疾病标志研究的发表文献数量为394篇。本文分析了2010-2024年间外泌体作为疾病生物标志物研究领域的发文情况,具体分析结果见图1,共计发文16674篇,总体发展呈逐年上升趋势。根据发文量的增长速度可将其发展趋势大致分为两个阶段,2010-2021年为第一阶段(快速增长期),该阶段外泌体的发文量呈指数型激增(指数拟合:*y*=77.796e^0.2855^*^x^*, *R*^2^=0.9984),根据发文量情况可以认为,2010-2016年是外泌体在生物标志物研究的初探时期;2017-2020年的增长速度逐渐稳定,发文量增幅在33%左右;2021-2024年为第二阶段(平稳期),该阶段的发文量皆超过2000篇,于2024年达到发文量顶峰(2519篇)。

**图1:**
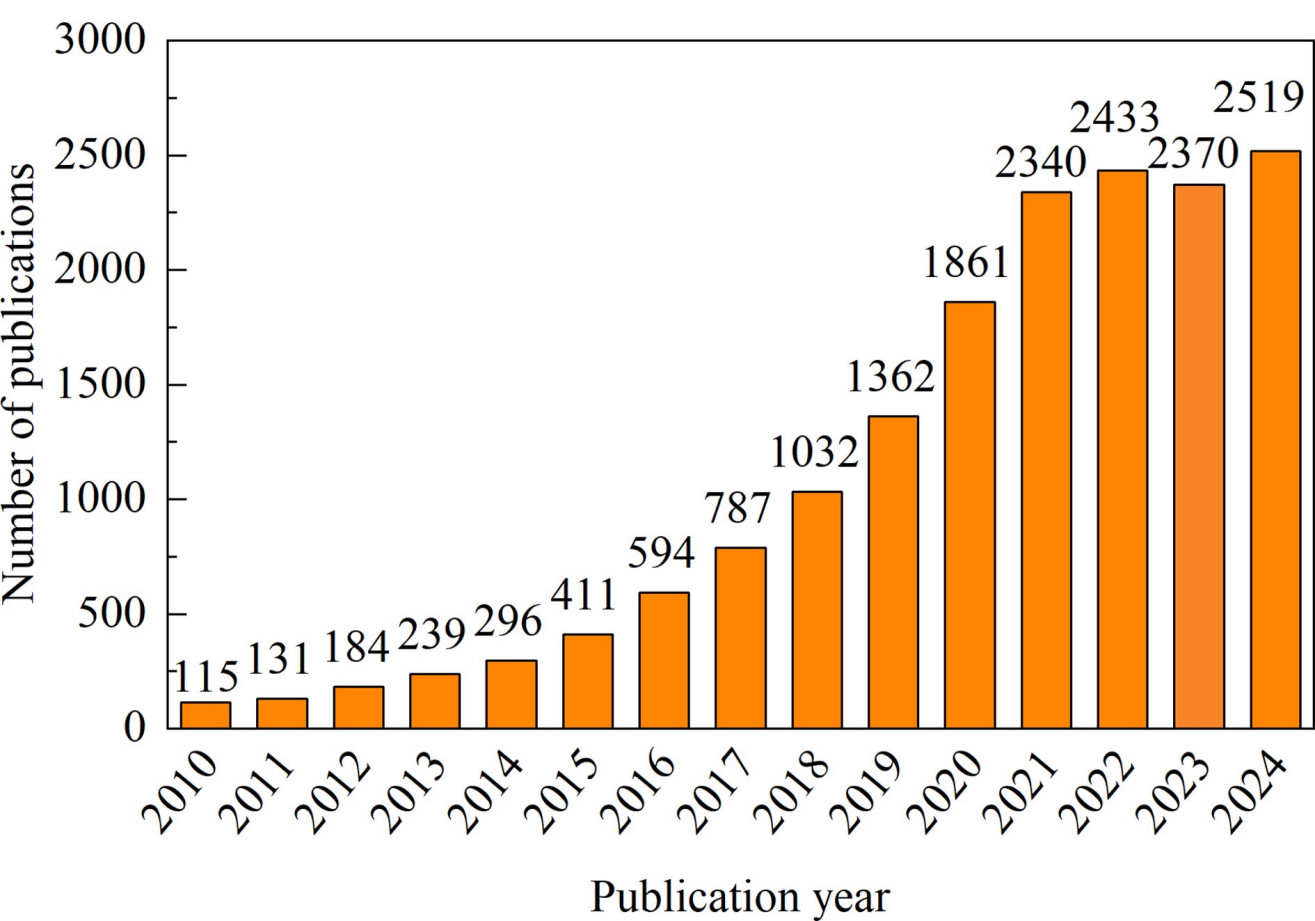
2010-2024年发文量分布

### 2.2 发文量与引用频次的分布

利用VOSviewer对检索得到的16674篇文献进行分析,其国家和机构分布见表1,中国的发文量最高,共计5873篇,占总发文量的35.2%;其次是美国(3987篇,23.9%)和意大利(1320篇,7.9%)。文献的被引频次可以反映其论文质量,被引频次越高在研究领域的影响力越大^[16]^。在引用频次上,澳大利亚和美国的篇均被引频次最高,平均57次/篇,然后是英国(48.32次/篇)。发文量在机构的分布上,上海交通大学、南京医科大学、复旦大学、中国科学院等国内科研机构共发文2145篇,占外泌体生物标志物研究领域的12.9%。哈佛医学院发文量为229篇,其篇均被引频次为64,是同比文献篇均被引频次最高的机构,说明其研究外泌体标志疾病的论文质量较高,影响力较大。

**表1:** 2010-2024年发文量排名前十的国家与机构

Country	Post volume	Percentage/ %	Average cited	Institution	Post volume	Percentage/ %	Average cited
China	6084	37.3	32	Shanghai Jiao Tong University	339	2.0	37
United States	3987	24.5	57	Nanjing Medical University	302	1.8	44
Italy	1320	8.1	38	Fudan University	290	1.7	33
Germany	969	5.9	46	Chinese Academy of Sciences	229	1.4	38
Spain	737	4.5	46	Harvard Medical School	229	1.4	64
Japan	733	4.5	41	Zhejiang University	208	1.2	39
England	703	4.3	48	Central South University	207	1.2	24
Korea	627	3.8	38	Southern Medical University	192	1.2	36
Australia	577	3.5	57	Shandong University	191	1.1	39
India	565	3.5	21	Capital Medical University	187	1.1	29

由VOSviewer分析文献发表期刊的发文量和被引频次可知,外泌体在生物标志物研究领域发文量排名前十的期刊平均影响因子为6.6,被引频次排名前十的期刊平均影响因子为9.8。发文量和引用频次前十的期刊共有13家,其中85%的期刊在JCR Q1,剩余15%在JCR Q2。

作者分布分析显示,东京医科大学的Ochiya发文量(69篇)和篇均被引频次(102次/篇)最高,其课题组主要从事外泌体介导的药物靶标和肿瘤标志物的相关研究。2018年,*Journal of Extracellular Vesicles*发表指南“Minimal information for studies of extracellular vesicles 2018 (MISEV2018): a position statement of the International Society for Extracellular Vesicles and update of the MISEV2014 guidelines”^[17]^;该指南基于ISEV于2014年发表的研究论文^[18]^,综合了2014-2018年的有关细胞外囊泡的研究信息,是一篇针对“细胞外囊泡”领域的研究指南。美国国立卫生研究院国家老龄化研究所(NIA)的学者Dimitrios共发文65篇,篇均被引频次为74,其研究课题关注阿尔茨海默病(Alzheimer’s disease, AD)的生物标记。

### 2.3 关键词分析

#### 2.3.1 关键词时间线图谱

文献的关键词融合了研究内容的核心点,对关键词做共现分析可以揭示学科研究的热点。关键词时间线图谱是在关键词聚类分析的基础上引入时间维度,能实现研究主题的多元、动态分析。利用Cite Space软件对WOSCC导出的文献数据作关键词时间线图谱,时间跨度为2010年1月至2024年12月,时间切片=1, g-Index *k*=3,分析结果如图2。图2中横轴表示时间,从左到右表示时间的推移;纵轴表示聚类,每一个聚类对应一个研究领域,并由不同颜色区分;时间线上的节点表示关键词,节点上的年轮代表关键词的使用年份(图中左下角标注各层年轮颜色代表的年份),节点越大,年轮越厚,关键词的出现频次越高。

**图2:**
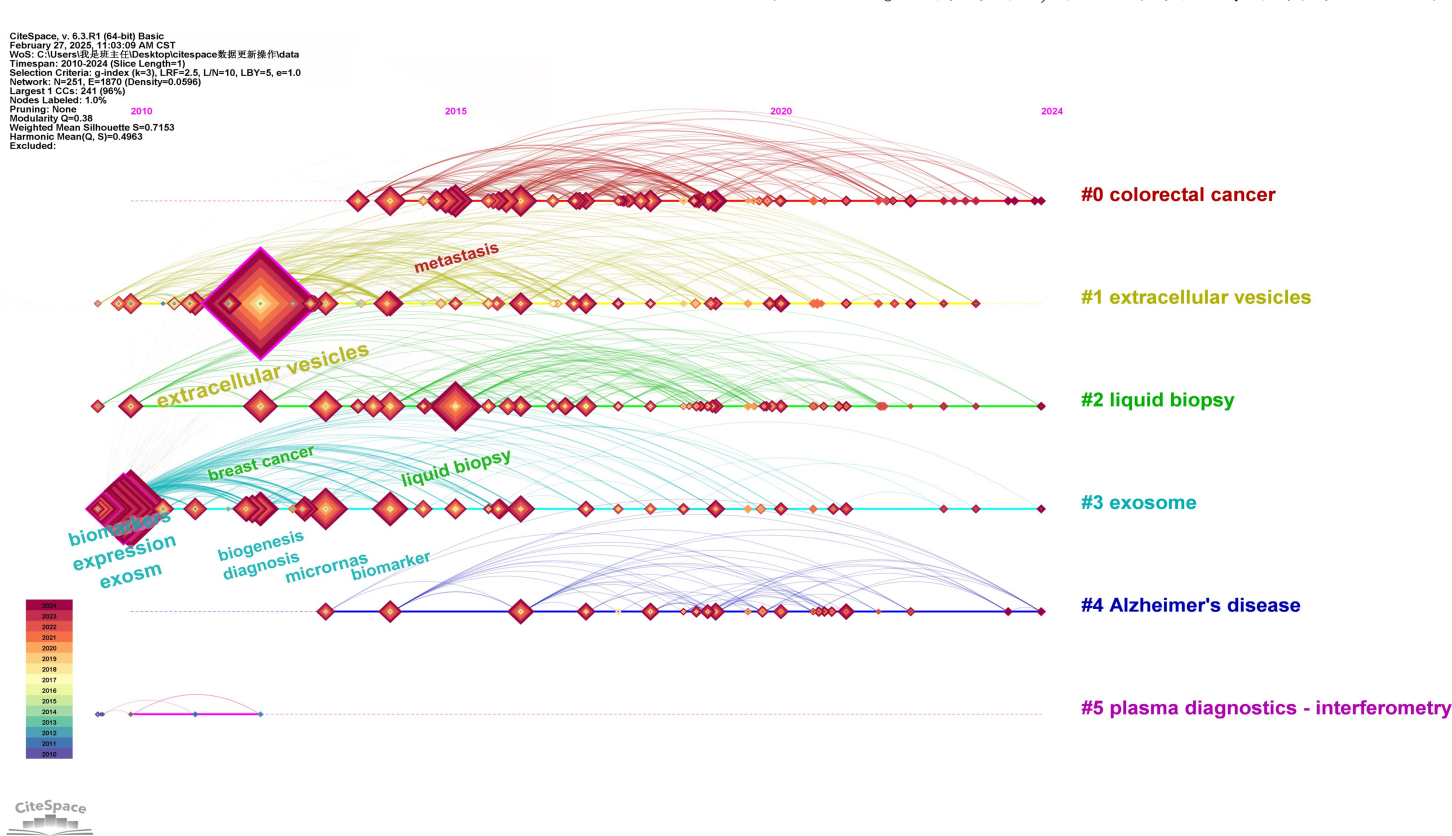
文献关键词在2010-2024年的时间线图谱

如图2所示,每个条线为一个聚类,分析共得出6个类别,分别为结直肠癌(colorectal cancer)、细胞外囊泡、液体活检、外泌体、AD、血浆诊断-干涉测量(plasma diagnostics-interferometry),见图2。结合关键词的聚类分析结果(见表2)分析,外泌体在生物标志物领域的研究热点主要集中在以下几个领域:(1)外泌体在以结直肠癌为代表的肿瘤微环境、信号通路、代谢、机体免疫与顺铂耐药性的研究,例如:携带miR-30a-5p的癌细胞源性外泌体能改变肿瘤相关血管的通透性,从而影响肝内胆管癌细胞的恶性转移^[19]^; (2)利用液体活检(尤其是aptasensor技术)高灵敏检测肿瘤源性的细胞衍生微粒(如外泌体、微泡等); (3)细胞外囊泡的蛋白质组学分析;(4)神经退行性疾病的早期诊断和治疗监测;(5)血浆诊断技术的优化和临床应用。

**表2:** 关键词聚类分析结果

Cluster	Keywords^*^
#0 colorectal cancer	miR-21; signaling pathway; metabolism; mesenchymal stem cell; long non-coding RNA therapeutic target; tumor associated macrophages; macrophage polarization; phenotype; outer membrane vesicles; non-coding RNAs; tumor suppressor; fibroblasts; noncoding RNA; chemoresistance; cisplatin resistance; NF kappa-B; promote; microRNA expression; diagnostic biomarker; RNAs; autophagy; squamous cell carcinoma; poor prognosis; macrophages; prognostic biomarker; long noncoding RNAs; mesenchymal transition; tumor-microenvironment; circular RNAs; up regulation; roles; potential biomarkers; inhibition; long noncoding RNA; cancer cells; noncoding RNAs; epithelial mesenchymal transition; pathway; drug resistance; carcinoma; cell proliferation; down regulation; promotes; migration; circular RNA; gastric cancer; apoptosis; tumor microenvironment; angiogenesis; resistance; invasion; growth; hepatocellular carcinoma; progression; colorectal cancer; proliferation; metastasis
#1 liquid biopsy	promote tumor growth; cell derived microparticles; human saliva; smooth muscle cells; rheumatoid arthritis; pregnancy; mesenchymal stromal cells; cancer therapy; serum exosome; endothelial microparticles; horizontal transfer; multivesicular body; urinary exosome; progenitor cells; responses; cancer exosome; tissue; heart failure; tissue factor; intercellular transfer; adipose tissue; tumor growth; emerging role; size; insulin resistance; nanoparticle tracking analysis; myocardial infarction; circulating microparticles; cardiovascular disease; differentiation; pathogenesis; intercellular communication; in vivo; messenger RNAs; flow cytometry; mediated transfer; stromal cells; nanoparticles; T cells; transferrin receptor; tumor derived exosome; endothelial cells; drug delivery; membrane vesicles; delivery; dendritic cells; proteomic analysis; gene expression; inflammation; microparticles; cell derived exosome; in vitro; stem cells; mesenchymal stem cells; extracellular vesicles
#2 exosome	aptasensor; amplification; heterogeneity; KRAS; exosomal miRNA; chip; women; cell-free DNA; biopsy; tumor cells; mortality; target; nucleic acids; precision medicine; clinical significance; recurrence; DNA methylation; cell lung cancer system; chemotherapy; early diagnosis; mutations; bladder cancer; cancer diagnosis; marker; potential biomarker; cell free DNA; separation; free DNA; classification; management; prognosis; circulating tumor DNA; quantification; peripheral blood; circulating exosome; diagnostic biomarkers; gene; ovarian cancer; risk; survival; blood; pancreatic cancer; messenger RNA; DNA; lung cancer; circulating tumor cells; prostate cancer; breast cancer; liquid biopsy
#3 extracellular vesicles	progress; glioblastoma; diabetic nephropathy; head; discovery; platform; proteomics; genes; reveals; fibrosis; injury; miRNA; extracellular vesicles (EVs); receptor; exosome isolation; biology; communication; mass spectrometry; miRNAs; release; therapy; markers; mechanisms; mechanism; vesicles; activation; disease; secretion; microRNA; RNA; extracellular vesicle; circulating microRNAs; proteins; protein; serum; biomarker; plasma; biogenesis; diagnosis; identification; microRNAs; cancer; microvesicles; cells; biomarkers; expression; exosome
#4 Alzheimer’s disease	cognitive impairment; neuronal exosome; Tau; children; infection; dementia; blood brain barrier; multiple sclerosis; binding; dysfunction; amyloid beta; central nervous system; model; cell; small extracellular vesicles; alpha synuclein; association; Parkinson’s disease; brain; oxidative stress; cerebrospinal fluid; Alzheimer’s disease
#5 plasma diagnostics-interferometry	deposition; density; spectroscopy and imaging; plasma diagnostics-interferometry; temperature; plasma diagnostics; diagnostics

* The order of clustering keywords is arranged from large to small according to frequency.

#### 2.3.2 关键词突现分析

突现词指的是一段时间内词频激增的关键词,关键词的突现分析可以反映领域研究的前沿。利用Cite Space的词频检测功能分析2010-2024年间的突现词,并展示爆发强度前30的突现词,结果如图3。图中突现词根据时间发展的顺序排列,由图可知,“circulating microRNAs”“microvesicles”“membrane vesicles”的爆发强度均大于80,关注时间为2010-2017年。其中“macrophages”“tumor growth”等词出现时间较晚,但爆发强度均大于20。总体来看,细胞外囊泡、肿瘤来源的外泌体、蛋白质组学分析、细胞间通信等已成为外泌体在生物标志物领域研究的经典主题;2015-2019年间的研究关注点转移为外泌体的分析技术、生物印记技术等,该阶段处于研究的稳步增长期;2020-2024年间的研究关注点转移到外泌体携带核酸、细胞自噬、肿瘤诊断等。通过突现词分析结果可以预测,外泌体作为疾病标志物的研究趋势是寻找更多外泌体标志物种类,探究标志物在疾病(尤其是肿瘤)各生理阶段的指示意义。

**图3:**
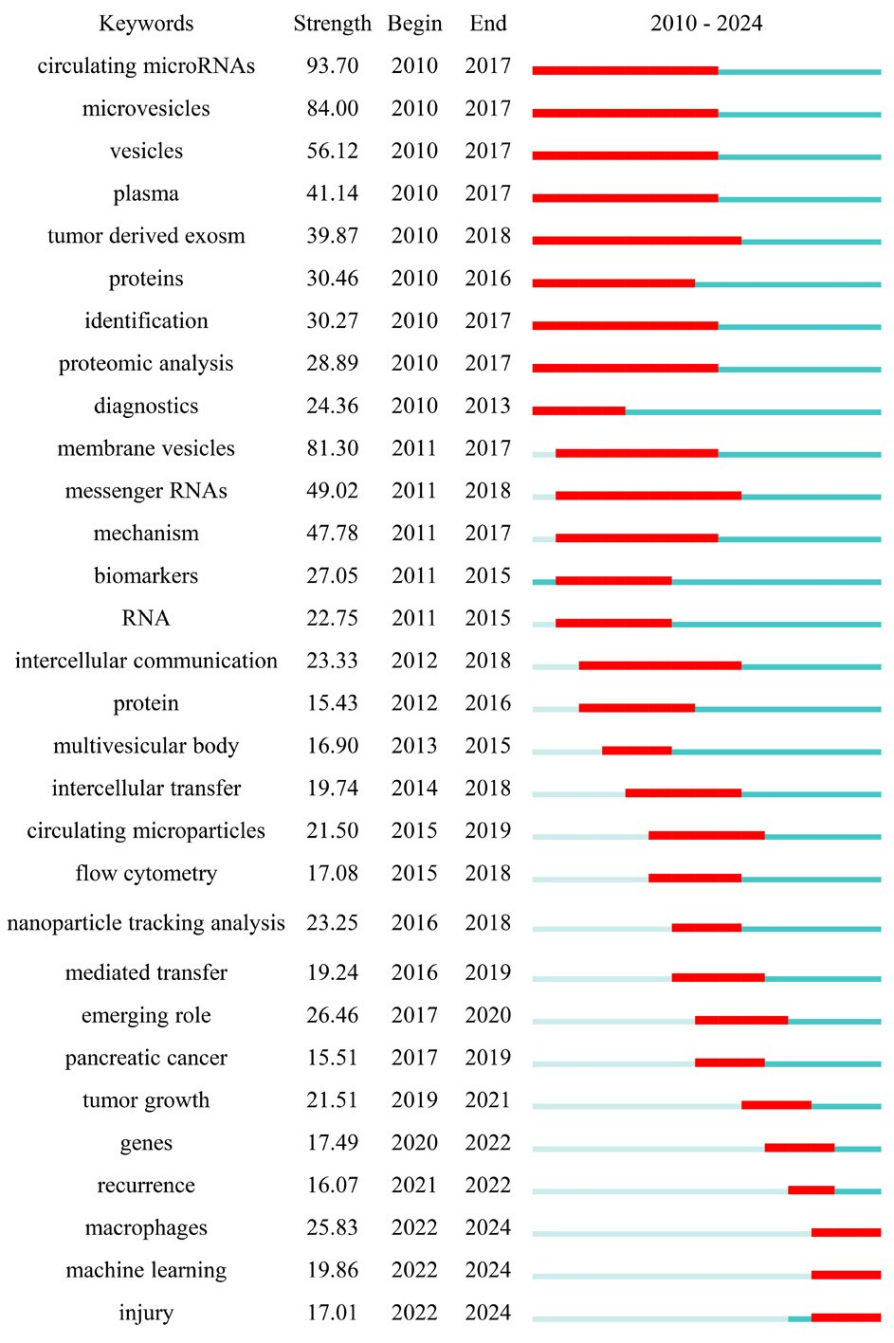
文献关键词在2010-2024年的突现性分析

### 2.4 共被引参考文献网络

文献共被引即两篇或多篇文献同时被后来发表的文献引用,通过文献共被引分析可以快速获知科学研究的发展情况。利用VOSviewer中的共被引网络分析功能,从521958篇共被引文献中筛选被引频次超过600的36篇文献,对其分析并分类,整理共被引文献中的高被引文献为代表性文献。

结果表明,参考文献的共被引分析结果主要分为3个聚类,表3列举了各个类群中的高被引文献。第一类文献的内容是1987-2013年间外泌体基础生理功能的研究;第二类文献的内容是2014-2021年间针对外泌体分离分析技术的研究;第三类是外泌体在肿瘤标记方面的研究。通过这3个类群可以知道,科学家们从发现外泌体到了解外泌体的生理调节机制,随后又积极探索开发外泌体的分离分析技术,目前的研究重心已经从实验室探究向临床疾病诊断治疗迁移。

**表3:** 共被引参考文献网络中各聚类中最有影响力的文献

Cluster	Author	Journal	DOI	Most influential paper	Citation
#1	Valadi H	*Nature Cell Biology*	10.1038/ncb1596	Exosome-mediated transfer of mRNAs and microRNAs is a novel mechanism of genetic exchange between cells	2620
	Raposo G	*Curr Opin Biotech*	10.1083/jcb.201211138.	Extracellular vesicles: exosomes, microvesicles, and friends	1840
	Skog J	*Nat Cell Biol*	10.1038/ncb1800	Glioblastoma microvesicles transport RNA and proteins that promote tumour growth and provide diagnostic biomarkers	1459
#2	Théry C	*J Extracell Vesicles*	10.1080/20013078.2018.1535750	Minimal information for studies of extracellular vesicles 2018 (MISEV2018): a position statement of the International Society for Extracellular Vesicles and update of the MISEV2014 guidelines	2380
	Kalluri R	*Science*	10.1126/science. aau6977.	The biology, function, and biomedical applications of exosomes	1788
	Van Niel G	*Nat Rev Mol Cell Bio*	10.1038/nrm.2017.125	Shedding light on the cell biology of extracellular vesicles	1492
#3	Melo S A	*Nature*	10.1038/nature14581	Glypican-1 identifies cancer exosomes and detects early pancreatic cancer	1176
	Hoshino A	*Nature*	10.1038/nature15756	Tumour exosome integrins determine organotropic metastasis	1115

## 3 文献研究内容

### 3.1 外泌体研究的分析技术

在WOSCC检索导出的16674篇文献中筛选出10042篇研究型文献“Article”,并使用关键词搜索,获得使用某关键词的文献数量。这种方法可以反映特定关键词的发文情况,初步掌握研究型论文的内容。

将分析技术大致分为4类:分子结构与组成分析、生物大分子分析、细胞与颗粒分析、形态学分析。搜索以各分析技术为论文关键词的文献,共搜索得到1093篇文献,结果如图4a。其中,分子结构与组成分析共434篇(39.7%),生物大分子分析共312篇(28.5%),细胞与颗粒分析共261篇(23.9%),形态学观察与成像技术共86篇(7.9%)。外泌体分析技术研究相关的文献数量占研究型文献的1/10,这说明外泌体作为生物标志物的研究重心是利用实验室成果来指导临床疾病诊疗。同时,研究内容的复杂性、关键词的多样性和文章数据的缺失等因素也不能忽略,关键词搜索的方式只能体现计量数据,不能完全与实际研究情况相同。

**图4:**
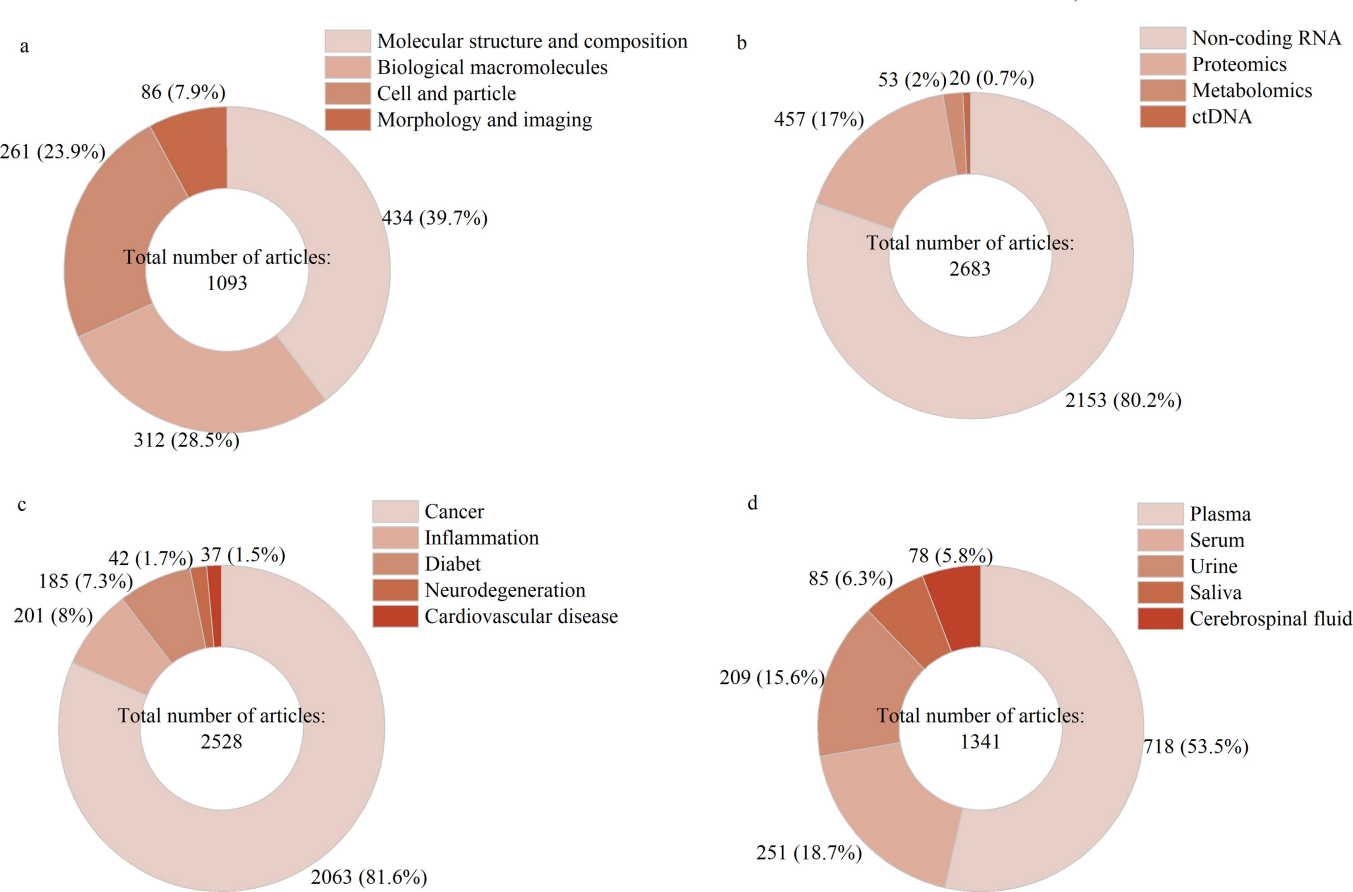
论文高频关键词的文章数量分布

#### 3.1.1 分子结构与组成分析

在434篇使用分子结构与组成分析技术的研究型文献中,141篇使用关键词“mass spectrometry”, 103篇使用关键词“chromatography”, 表面增强拉曼光谱技术(surface-enhanced Raman spectroscopy,SERS)被广泛采用,85篇使用关键词“Raman”(其中使用关键词“SERS”的文献有52篇),82篇使用关键词“electrochemical”, 23篇使用关键词“surface plasmon resonance”。通过数据结果可以知道,在外泌体研究中,通常使用色谱法、质谱法、拉曼光谱技术和电化学分析技术来分析外泌体中的复杂分子,而核磁共振分析、表面等离子共振技术的使用相对较少。下面将简单介绍这些技术在外泌体作为疾病生物标志物的应用。

外泌体分离分析中最常使用液相色谱法,其中体积排阻色谱、亲和色谱、离子交换色谱模式广泛用于外泌体的富集^[20,21]^。在103篇以“chromatography”为关键词的研究型论文中,同时使用“size exclusion chromatography”的文献有41篇,可见体积排阻色谱法是最常用的外泌体分离分析相关的色谱技术。

质谱是外泌体蛋白质组学的主流分析手段,外泌体相关的分析方式包括气相色谱-质谱联用和液相色谱-质谱联用。例如Montaldo等^[22]^对39例慢性丙肝感染且经历持续病毒学反应后的患者采血纯化,使用纳升液相色谱-串联质谱(nanoLC-MS/MS)技术分析EVs中的纤维化信号,蛋白质组学结果显示健康个体和慢性丙肝感染者的蛋白表达水平有显著差异,其中纤维化蛋白DIAPH1显著上调;Moraes等^[23]^采集重症COVID-19患者血样,并使用nanoLC-MS/MS技术分析EVs蛋白质丰度变化,蛋白质组学结果显示,重症患者均出现C反应蛋白、纤维蛋白原和D二聚体的循环水平升高;Surman等^[24]^使用nanoLC-MS/MS技术分析人正常甲状腺滤泡上皮细胞和人甲状腺间变性癌细胞条件培养基中的外泌体包裹肽段,发现两种细胞的外泌体携带蛋白质量有显著差异,且癌变细胞分泌出538种特殊蛋白质。结合以上研究发现,质谱技术已经广泛运用在外泌体标记疾病的蛋白质组学分析中,成为一种病变指标检测的常用技术。

拉曼光谱法是基于拉曼效应的一种结构分析技术,当光照射在物质表面上并发生非弹散射时,其散射光谱与入射光的频率发生改变,可以获得物质分子振动和转动频率的信息,依此计算分子的结构^[25]^。然而经典的自发拉曼光谱信号较弱,其应用因此受限,实验中常使用SERS分析技术,即金属纳米结构增强拉曼信号。21世纪始,SERS技术已成为一项高效的振动光谱和成像技术,基于SERS的液体活检技术有望成为实现精准医疗的强大工具。Qin等^[26]^受海洋藤壶吸附游泳鲸鱼现象和微观纳米蛋白冠结构的启发,开发了一种以自驱动固态微气泡为模板,聚乙烯亚胺纳米层作为“藤壶胶”,金属有机框架(MOF)高密度组装的纳米冠。该研究利用MOF中的Z ${r_{4}}^{+}$ 离子与细胞外囊泡膜配位识别,耦合双编码SERS技术,能快速分离细胞外囊泡,并且支持多元SERS分析,检出限低,研究结果可以为临床癌症诊断和治疗提供一种液体活检新思路。同时,Shin等^[27]^使用基于深度学习的外泌体SERS技术做肺癌早期诊断,发现深度学习模型能区分正常和肺癌细胞系的外泌体(准确率为95%),并预测患者血浆外泌体与肺癌细胞外泌体的相似性高于正常人水平,说明外泌体检测与深度学习的结合有着巨大的发展潜力。

电化学法是在加电场的化学池中,离子经过氧化还原反应,其电子在固体电极和溶液之间转移的现象^[28]^。Sun等^[29]^利用环切口酶和杂交链式反应扩增信号,检测含有外泌体表面适配体的发夹适体探针,当适配体与蛋白质酪氨酸激酶7(PTK7)特异结合时探针构象改变。这种方法可以巧妙地通过DNA检测来检测外泌体。同时这一信号放大的结果能有效检测体液中的肿瘤源性外泌体,证实其在临床诊断上的发展潜力。

#### 3.1.2 生物大分子分析

外泌体内容物主要有核酸、蛋白质、磷脂、表面多糖等^[2]^。在312篇使用生物大分子分析技术的研究型文献中,针对核酸类内容物的分析技术有62篇使用关键词“microarray”, 60篇使用关键词“RNA-seq”, 59篇使用关键词“next generation sequencing”, 33篇使用关键词“qRT-PCR”, 25篇使用关键词“CRISPR”, 22篇使用关键词“qPCR”, 10篇使用关键词“dPCR”;针对蛋白质类内容物的分析技术中,38篇使用关键词“ELISA”, 3篇使用关键词“immunofluorescence”。通过关键词使用频次可以推测,关注外泌体核酸的研究数量多于外泌体蛋白,该推测与图4b的数据结果相吻合。值得关注的是,自2020年始,学者们尝试将基因编辑技术CRISPR与外泌体核酸和蛋白质信号检测技术联合,制造疾病诊断传感器,目前CRISPR-Cas12a是主要使用的基因编辑系统。

#### 3.1.3 细胞与颗粒分析

261篇使用细胞与颗粒分析技术的文献中,132篇使用关键词“flow cytometry”, 73篇使用关键词“microfluidics”, 45篇使用关键词“nanoparticle tracking analysis”, 11篇使用关键词“dynamic light scattering”。这些数据显示,流式细胞术在外泌体分析中占较大比重。结合关键词突现分析(见图3)知,流式细胞术是2015-2018年间的研究热点。流式细胞术是外泌体分析的一项重要技术,可以对单个细胞及囊泡计数分析并分选。纳米粒子追踪和动态光散射技术都是基于粒子布朗运动原理的颗粒分析技术,且纳米粒子追踪可以对检测物中的粒子浓度进行计算^[30]^。

#### 3.1.4 形态学观察与成像技术

同时,有86篇文献利用显微技术来观察外泌体形态,31篇使用关键词“electron microscopy”, 24篇使用关键词“magnetic resonance”, 20篇使用关键词“atomic force microscopy”, 8篇使用关键词“fluorescence microscopy”, 3篇使用关键词“fluorescence imaging”。其中透射电镜技术、扫描电镜技术和冷冻电镜技术使用频率较高。

总体来说,外泌体及其内容物分析技术的发展已经成熟,同时仍有学者致力于优化改善经典分析技术,期望达到降低分析检测成本,提高检测灵敏度和精确度的目的。

### 3.2 外泌体标志物类型

搜索以“ctDNA”“microRNA”“miRNA”“lncRNA”“circRNA”“proteomics”“metabolomics”为论文关键词的文献,结果如图4b。共搜索得到2683篇研究型文献,使用关键词“microRNA”“miRNA”“lncRNA”“circRNA”的文献共2153篇(80.2%),这些RNA均属于非编码RNA (non-coding RNA)。其中,1074篇使用关键词“microRNA”, 815篇使用关键词“miRNA”, 157篇使用关键词“lncRNA”, 107篇使用关键词“circRNA”。此外,457篇(17.0%)使用关键词“proteomics”; 53篇(2.0%)使用关键词“metabolomics”; 20篇(0.7%)使用关键词“ctDNA”。这些数据反映,外泌体非编码RNA和蛋白质组学研究备受关注。根据词频统计结果得知,目前miR-21、miR-1246、miR-1290、miR-181a-5p等microRNA是疾病标志的主要研究对象,miR-21是外泌体microRNA的研究热点,大多数癌细胞高表达miR-21,其异常水平影响癌症进展和DOX耐药,在癌症治疗中十分关键^[31]^。外泌体蛋白中的肿瘤相关蛋白(PD-L1、EGFR、EpCAM)、神经细胞相关蛋白(*β*-淀粉样蛋白、*α*-突触核蛋白、Tau蛋白等)、CD蛋白(CD63、CD9、CD8、CD44等)关注度较高。

代谢组学和ctDNA标志物的研究较少,这主要是因为细胞代谢物和ctDNA在体液中的含量极少,在检测时需要大量样本并且需要高灵敏度的检测仪器捕获标志物,增加检测成本,所以没有广泛应用在临床检测^[2,32]^。Jones等^[33]^认为使用脑脊液替代血浆可以避免非肿瘤细胞产生的大量游离DNA,获得较高浓度的ctDNA,这可以为ctDNA标记肿瘤的研究提供新思路。值得注意的是,汇集多研究组织的代谢组学结果,分析癌症细胞代谢物差异水平或者重叠类群对癌症生长的代谢机制探索有巨大的研究潜力。

### 3.3 疾病类型

搜索以“cancer”“inflammation”“neurodegeneration”“cardiovascular disease”“diabet”为论文关键词的文献,结果如图4c。共搜索得到2528篇文献,其中,2063篇(81.6%)使用关键词“cancer”, 201篇(8.0%)使用关键词“inflammation”, 185篇(7.3%)使用关键词“diabet”; 42篇(1.7%)使用关键词“neurodegeneration”, 37篇(1.5%)使用关键词“cardiovascular disease”。根据数据可以知道,外泌体在疾病标志物研究中聚焦于癌症诊断和治疗,同时有少数学者关注外泌体内容物标志炎症(如骨关节炎、类风湿性关节炎)、糖尿病(如胰岛素抵抗、糖尿病肾病)、神经退行性疾病(如阿尔茨海默病、帕金森病、肌萎缩侧索硬化症等)、心血管疾病(如心肌梗死、急性心肌梗死、心力衰竭、动脉粥样硬化等)等疾病。

聚焦分析癌症相关的研究领域,以“cancer”为关键词的2063篇研究论文中使用关键词搜索的结果如下:“lung cancer”共285篇,“breast cancer”共274篇,“prostate cancer”共231篇,“colorectal cancer”共214篇,“gastric cancer”共140篇, “pancreatic cancer”共128篇,“ovarian cancer”共118篇,“bladder cancer”共73篇。该结果说明,多数学者关注外泌体标志肺癌、乳腺癌、前列腺癌以及结直肠癌等8种高发癌症。

### 3.4 样本类型

搜索以“plasma”“serum”“urine”“saliva”以及“cerebrospinal fluid”为论文关键词的文献,结果如图4d。共搜索得到1341篇文献,其中,718篇(53.5%)使用关键词“plasma”, 251篇(18.7%)使用关键词“serum”, 209篇(15.6%)使用关键词“urine”, 85篇(6.3%)使用关键词“saliva”, 78篇(5.8%)使用关键词“cerebrospinal fluid”。根据数据,疾病诊断和治疗中血液样本(包括血浆和血清)最常用,其次是尿液样本,唾液和脑脊液尚未广泛应用于各个指标的检测中。目前,癌症外泌体检测主要使用血液样本和尿液样本,通过microRNA和特定蛋白质的表达水平来判断细胞是否发生癌变;炎症的液体活检主要使用血液样本,经典炎症标志主要有C反应蛋白水平、红细胞沉降率和降钙素原水平,而泌尿系统炎症诊断主要采集尿液样本^[34]^;糖尿病的液体活检和治疗主要采集血液和尿液样本^[35,36]^。外泌体microRNA的液体活检是阿尔茨海默病和帕金森病等神经退行性疾病的主要检测手段^[37]^。外泌体在动脉粥样硬化早期的促炎作用使其在心血管疾病中受到关注,目前在心血管疾病诊断治疗的研究尚处于起步阶段^[38]^。

## 4 总结

本文利用文献计量学方法对外泌体在生物标志物领域研究的文献背景信息、文献关键词、共被引参考文献做分析。从全球发文量数据来看,中国学者在外泌体作为生物标志物的研究中发挥主要作用;高发文量期刊和高被引期刊的影响因子较高,多数为JCR Q1分区期刊,对外泌体相关研究有重要的指导意义;关键词聚类的时间线分布显示外泌体作为疾病标志物的研究主要关注癌症、蛋白质组学分析、神经退行性疾病、液体活检等话题;文献突现词从组学分析、代谢通路等内容向肿瘤标记转变;研究框架主要是外泌体生理功能、外泌体分析技术、外泌体疾病标志3大类,其中肿瘤标志关注度最高。

从研究内容上看,外泌体标记疾病的研究热点是microRNA,尤其是癌症诊断和治疗。也有不少学者从事外泌体蛋白质组学分析的研究,外泌体蛋白质组学分析常用技术包括色谱、质谱、拉曼光谱和电化学传感等。外泌体携带蛋白质可以反映神经退行性疾病的病理状况,尤其是*β*-淀粉样蛋白、Tau蛋白和*α*-突刺核蛋白等。液体活检技术搭建了外泌体作为生物标志物的理论研究与实践应用的桥梁,在临床检验时通常采集血液、尿液样本送检。

综上所述,过去15年完成了外泌体作为生物标志物的基础研究工作,外泌体分离分析技术已经成熟并不断改善创新。现阶段处于研究的平稳期,研究的关注点转为肿瘤诊断、细胞自噬、分离分析技术优化等主题。
